# Does Ethnicity Affect Where People with Cancer Die? A Population-Based 10 Year Study

**DOI:** 10.1371/journal.pone.0095052

**Published:** 2014-04-21

**Authors:** Jonathan Koffman, Yuen King Ho, Joanna Davies, Wei Gao, Irene J. Higginson

**Affiliations:** King's College London, Cicely Saunders Institute, Department of Palliative Care, Policy and Rehabilitation, London, United Kingdom; Hormel Institute, University of Minnesota, United States of America

## Abstract

**Background:**

Ageing is a growing issue for people from UK black, Asian and minority ethnic (BAME) groups. The health experiences of these groups are recognised as a ‘tracer’ to measure success in end of life patient-preferred outcomes that includes place of death (PoD).

**Aim:**

To examine patterns in PoD among BAME groups who died of cancer.

**Material and Methods:**

Mortality data for 93,375 cancer deaths of those aged ≥65 years in London from 2001–2010 were obtained from the UK Office for National Statistics (ONS). Decedent's country of birth was used as a proxy for ethnicity. Linear regression examined trends in place of death across the eight ethnic groups and Poisson regression examined the association between country of birth and place of death.

**Results:**

76% decedents were born in the UK, followed by Ireland (5.9%), Europe(5.4%) and Caribbean(4.3%). Most deaths(52.5%) occurred in hospital, followed by home(18.7%). During the study period, deaths in hospital declined with an increase in home deaths; trend for time analysis for those born in UK(0.50%/yr[0.36–0.64%]p<0.001), Europe (1.00%/yr[0.64–1.30%]p<0.001), Asia(1.09%/yr[0.94–1.20%]p<0.001) and Caribbean(1.03%/yr[0.72–1.30%]p<0.001). However, time consistent gaps across the geographical groups remained. Following adjustment hospital deaths were more likely for those born in Asia(Proportion ratio(PR)1.12[95%CI1.08–1.15]p<0.001) and Africa(PR 1.11[95%CI1.07–1.16]p<0.001). Hospice deaths were less likely for those born in Asia(PR 0.73 [0.68–0.80] p<0.001), Africa (PR 0.83[95%CI0.74–0.93]p<0.001), and ‘other’ geographical regions (PR0.90[95% 0.82–0.98]p<0.001). Home deaths were less likely for those born in the Caribbean(PR0.91[95%CI 0.85–0.98]p<0.001).

**Conclusions:**

Location of death varies by country of birth. BAME groups are more likely to die in a hospital and less likely to die at home or in a hospice. Further investigation is needed to determine whether these differences result from patient-centred preferences, or other environment or service-related factors. This knowledge will enable strategies to be developed to improve access to relevant palliative care and related services, where necessary.

## Introduction

Globalisation has brought rapidly increasing numbers of black, Asian and minority ethnic (BAME) populations who have migrated to developed countries [1]. In 2005, there were an estimated 191 million immigrants across the globe: approximately 64 million of these immigrants were in Europe and 44 million in North America, a tripling of immigrant populations in these regions compared to twenty years ago [1]. This trend is expected to continue [2]. One characteristic of ethnic minority populations in Europe is that they are not evenly distributed and often concentrate in cities. For example, within the United Kingdom, London is the most ethnically diverse region with the lowest proportion (59.8%) of people who identify as being White British [3]. Migration and ethnicity represent two closely interrelated phenomena that are associated with major differences in environment and culture, and are regarded as being constituent components of ethnicity [4] [5].

In an ageing population such as the United Kingdom's (UK), cancer affects an increasing number of people from all ethnic backgrounds and it is important to understand variation in outcomes. However, despite universal health coverage with free access to NHS services and a largely voluntary-aided hospice sector, a mixed picture emerges in relation to how those from BAME groups access, and experience, health services in general [6] [7], during cancer care [8], [9], and at the end of life [10] [11]. One of the highest priorities for public health research, policy and practice is to reduce inequalities. This is achieved not through passive convergence, but by improving opportunities to enhance health outcomes, life expectancy, heath-related quality of life and quality of health care of the less priviledged groups, including those from BAME groups, so that they converge with those of the majority population [4] [12].

Furthermore, the experience of older members from Black, Asian and minority ethnic (BAME) groups is increasingly recognised as a crucial ‘tracer’ for measuring the success in achieving health and patient-preferred outcomes for the population in general [8], and specifically at the end of life [13]. We therefore chose to focus on one common important outcome, place of death; which is judged by patients, their families, health professionals, policy makers and researchers to be a central issue [14] [15] [16] [17]. In this paper, we investigate whether place of death for those who died in London from all cancer causes differed according to geographical origin (i.e. country of birth) and over time.

## Method

### Design

Population-based study of cancer deaths in London from 2001–2010.

### Ethics and permission

Following ONS procedures a Data Access Agreement was signed in a formal data management agreement. All researchers accessing the data (WG, IJH, HK and JMD) were approved by ONS. This study was based on fully anonymised records, therefore no ethical approval was required according to the Information Commissioner's Office guidelines, ONS procedures and King's College London Research Ethics Committee.

### Setting

We focused on London, the most ethnically diverse city in the UK and one of the most ethnically diverse cities in the world [18] to ensure large enough proportions of BAME people were captured in the dataset to strengthen the analysis. Rural areas in the UK tend to have very high proportions of UK born residents (94.9% compared to 84.7% on average across all urban areas in 2011). London is the UK capital city comprising 8.2 million people [19], it is an example of an urban setting with a relatively high proportion of BAME residents [3]. In 2001 and 2011 London had the largest proportion of foreign born residents compared to other regions in the UK, increasing by 10% from 27% (1.9 million) in 2001 to 37% (3.0 million) in 2011 [19]. More than one in three usual residents in London is non-UK born, with above national average proportions for most BAME groups including those identified as being African (7.0%), Indian (6.6%), and Caribbean (4.2%). London also had the highest proportion of residents recorded as ‘any other white’ (12.6%) compared to other UK regions [20]. Whilst the White British population remains the largest ethnic group (44.9%) in London, this proportion is lower than other areas in England and Wales [20].

### Study population and data sources

Mortality data for all deaths in England 2001–2010 were obtained from the UK Office for National Statistics (ONS). Death registration is the most useful source of national data on cause and place of death [17]. By law in England, a death must be registered within five days, unless it becomes the subject of a coroner's inquiry. The quality of the information is considered to be very high quality for cancer [21] and a very small number of causes of death remain legally unclassified (less than 0.16% in 2002) [22]. Therefore, these data allow for whole population analysis and for national and international comparisons [23]. The information recorded on the death registration certificate by the medical practitioner includes (i) cause of death; (ii) age of the decedent, (iii) date and place of death, and (iv) other information obtained by the Registrar's Office at the time of death registration including marital status, address of residence and country of birth. Death registry data in the UK does not currently record ethnicity. The decedent's country of birth is a pragmatic proxy for ethnicity [6] [24] [25] [26], limited to first generation migrants.

### Inclusion and exclusion criteria

The sample included those aged ≥65 years, to capture first generation migrants born outside of the UK, and exclude second or third generation migrants born in the UK, who died from cancer. Our sample included those born in the UK, expected to be mostly White British, and first generation migrants from Ireland, Europe, Asia, the Caribbean, Africa, China and ‘other locations’ considered to be consistent over time [27] [28].

A subset of the individual-level death registration data from 2001–2010, as collected by the ONS, were used in this study. Inclusion criteria included (i) cancer as the underlying cause of death (ICD-10 codes C00-C97); (ii) deaths for those aged ≥65 years; (iii) we excluded 2006 from the study due to an ONS coding change rendering it impossible to reliably extract country of birth from the codes.

### Study variables

Variables included: age, gender, year of death, marital status, cancer type, and country of birth. Country of birth was aggregated into eight groups: UK, Ireland, Europe, Asia, the Caribbean, Africa, China, and ‘other’. Countries categorised as ‘other’ accounted for <3% of records. Place of death was grouped into four categories: home, hospital, hospice and other communal establishments (including nursing home, residential home and care homes). Hospice refers to a dedicated unit with in-patient beds; these are usually freestanding from hospitals. An area-based measure of deprivation was assigned to individuals in the dataset using the Index of Multiple Deprivation 2010 (IMD 2010) [29], linked at Lower Super Output Area level to the residential postcode of the deceased and grouped according to national quintiles; with 1 being most deprived and 5 being least deprived.

### Statistical analysis

Statistical changes over time in the proportion of people dying at home, hospital, hospice or other communal establishments, for the period 2001 to 2010, were tested for using linear regression. Poisson regression with robust variance was used to estimate the association between country of birth and place of death, measured using proportion ratio (PR). Four models were developed including: (i) deaths in hospital versus deaths in all other locations; (ii) deaths in hospice versus deaths in all other locations; (iii) deaths at home versus deaths in all other locations; and (iv) deaths in communal establishments versus deaths in all other locations. In all models, the group born in the UK was used as the baseline. The association was adjusted for sex, age, year, marital status, IMD 2010 quintile and cancer type. Due to the very small number of cases within the Chinese group, these cases were combined with the larger and geographically proximal, ‘Asian’ group for time trend and Poisson regression analyses. In the case of over-disperation or under-disperation in the response variable, alternative models (negative and quasi-poission models) were tested and comaprted to the Poisson regression model.

All analyses were performed using R version 2.15.1 (R Foundation for Statistical Computing, Vienna, Austria).

## Results

A total of 93,375 people aged ≥65 years died from cancer between 2001 and 2010 in London (Table 1). Their mean age was 78.5 years (SD 7.7 years). Over half were men (51.9%) and the principal causes of death were from lung cancer (22.5%) and colorectal cancers (10.3%). Most deaths were in hospitals (52.5%, n = 49,032) followed by home (18.7%, n = 17,445). A total of 76% (n = 70,951) of all of those who died, were UK born. The largest groups born outside the UK originated from Ireland (5.9%, n = 5,507), Europe (5.4%, n = 5,069) and the Caribbean (4.3%, n = 4,059). Over the study period, 24.8% of all cancer deaths in London were from people living in the most deprived areas, compared to 10.3% from people living in the least deprived areas.

**Table 1:** Demographic characteristics of all cancer deaths in London from 2001–2010.

Variable	Number of deaths	%
**Gender:**		
Male	48,495	51.9%
Female	44,880	48.1%
**Marital status:**		
Unknown	1,025	1.1%
Single	9,843	10.5%
Married	39,597	42.4%
Divorced	7,565	8.1%
Divorced	35,345	37.9%
**Place of death:**		
Hospice	16,972	18.2%
Own residence	17,445	18.7%
Hospital	49,032	52.5%
Other communal	8,757	9.4%
Elsewhere	1,169	1.3%
**Index of multiple deprivation (IMD 2010):**		
1 (most deprived)	23,133	24.8%
2	26,197	28.1%
3	18,680	20.0%
4	15,752	16.9%
5 (least deprived)	9,613	10.3%
**Cancer cause of death:**		
Bladder	3,323	3.6%
Breast	6,656	7.1%
Colorectal	9,587	10.3%
Haematology	7,559	8.1%
Head & Neck	1,543	1.9%
Kidney	1,770	1.9%
Liver	2,083	2.2%
Lung	21,031	22.5%
Oesophagus	3,635	3.9%
Ovarian	2,273	2.4%
Pancreas	4,659	5.0%
Prostate	7,558	8.1%
Stomach	3,668	3.9%
Others	18,030	19.3%
**Region of birth:**		
UK	70,951	76.0%
Ireland	5,507	5.9%
Europe	5,069	5.4%
Asia	3,441	3.7%
Caribbean	4,059	4.3%
African	1,543	1.7%
Chinese	290	0.3%
Other	2,515	2.7%
**Mean age (years, SD):**		
UK	79.1	7.8
Ireland	76.7	7.3
Europe	78.7	7.7
Asia	75.8	6.8
Caribbean	75.3	6.8
African	73.8	6.6
Chinese	76.5	7.3
Other	76.6	7.5

**Figure 1:**
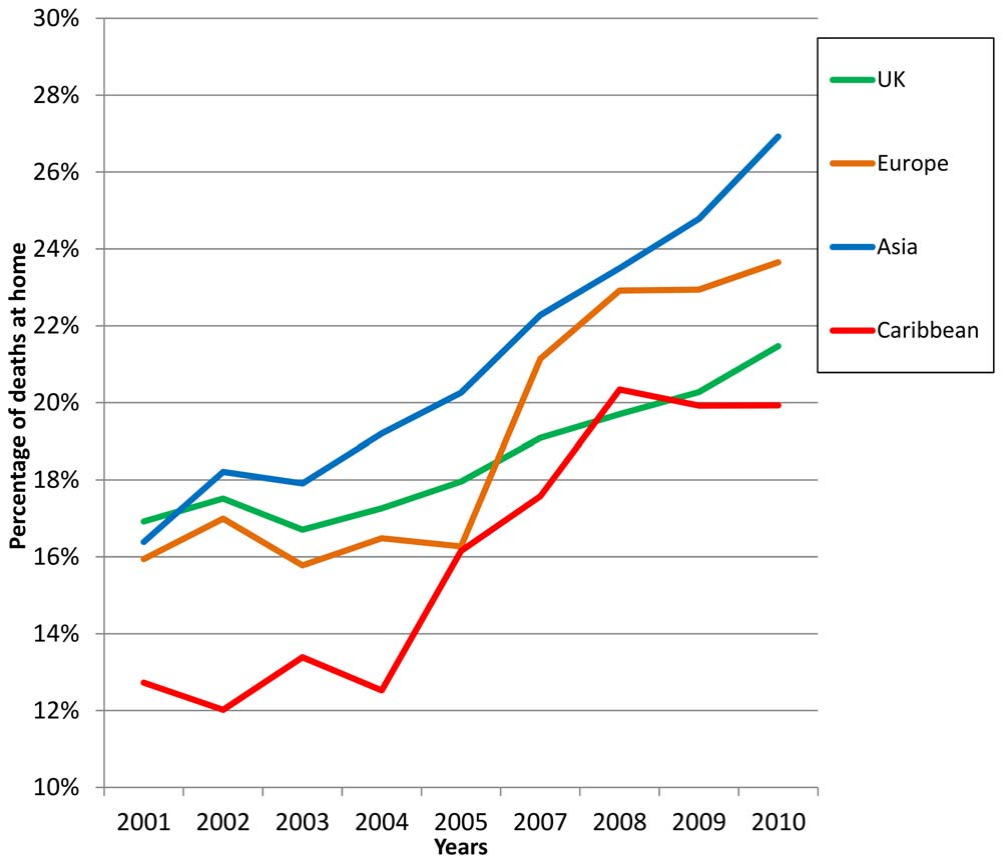
Percentage of deaths at home across London from 2001–2010 by region of birth.

**Figure 2:**
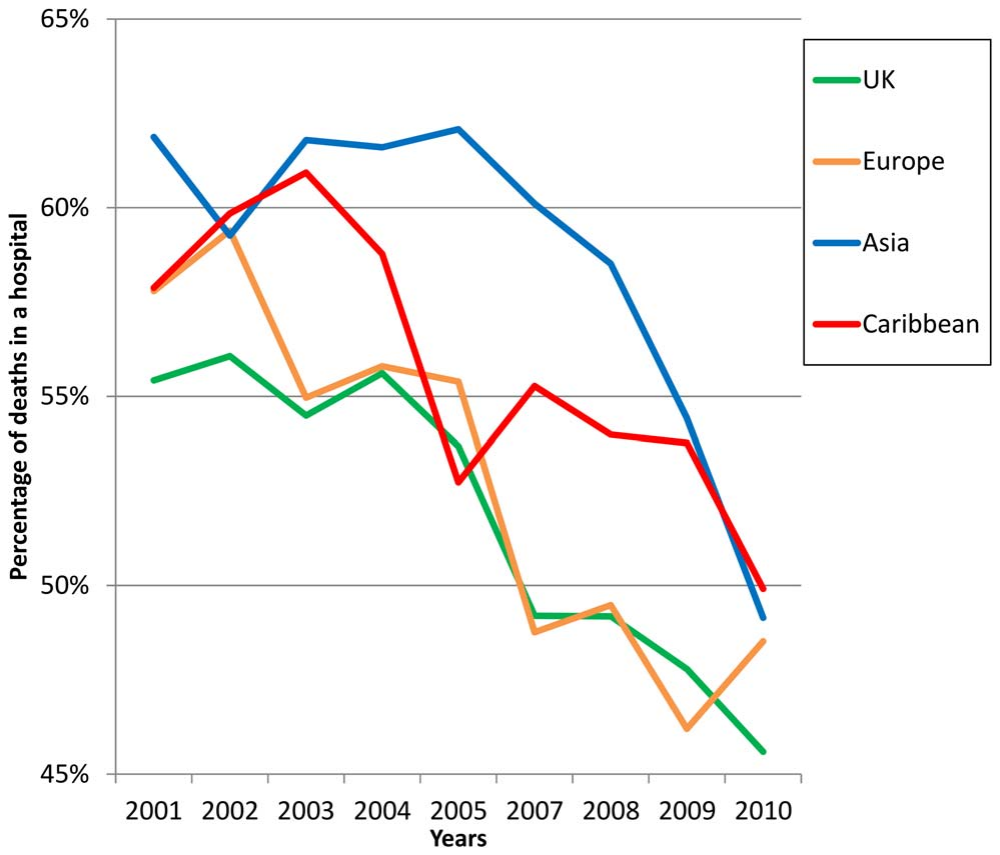
Percentage of deaths in a hospital across London from 2001–2010 by region of birth.

For hospice deaths, the proportion of deaths among those born in the UK, Ireland and Europe remained relatively stable over time; analyses of trends over time did not detect any statistically significant changes (Table 2). The proportion of deaths at home steadily increased for all groups (figure 1), however, gaps across the groups according to where people were born were evident and did not diminish over the study period. Although the proportion of hospital cancer-related deaths in London for those from all groups declined over the study period (figure 2), we observed broadly consistent gaps according to where people were born. With the exception of those originating from Africa, these reductions were statistically significant and were most prominent for four groups originating from Europe, the UK, Asia and the Caribbean.

**Table 2:** Location of death among all cancer deaths in London by region of birth (2001–2010).

(%, 95% CI)									
Place of Death	UK (n = 70,951)	Ireland (n = 5,507)	Europe (n = 5,069)	Asia (n = 3,441)	Caribbean (n = 4,059)	African (n = 1,543)	Chinese (n = 290)	Others (n = 2,515)	Total (n = 93,375)
Hospice	18.1 (CI 17.8–18.4)	20.3 (CI 19.2–23.3)	19.3 (CI 18.2–20.4)	13.9 (CI 12.7–15)	19.9 (CI 18.7–21.1)	16.7 (CI 14.9–18.6)	16.9 (CI 12.6–21.2)	17.1 (CI 15.6–18.6)	18.2 (CI 17.9–18.4)
Own Residence	18.4 (CI 18.2.18.7)	20.7 (CI 19.7–21.8)	19.2 (CI 18.1–20.3)	21.5 (CI 20.1–22.9)	16.6 (CI 15.4–17.7)	18.9% (CI 17.0–20.9)	13.8 (CI 9.8–17.8)	20.0 (CI18.4–21.6)	18.7 (CI 18.4–18.9)
Hospital	52.1 (CI 51.8–52.5)	48.5 (CI 47.2–49.8)	52.8 (CI 51.5–54.2)	58.3 (CI 56.6–59.9)	55.5 (CI 53.9–57.0)	58.3% (CI 55.9–60.8)	63.8 (CI 58.3–69.3)	53.6 (CI 51.6–55.5)	52.5 (CI 52.2–52.8)
Other Communal	10.0 (CI 9.8–10.2)	9.1 (CI 8.4–9.9)	7.7 (CI 7.0–8.4)	4.7 (CI 4.0–5.4)	7.3 (CI 6.5–8.1)	5.1 (CI 4.0–6.1)	4.5 (CI 2.1–6.9)	8.0 (CI 7.0–9.1)	9.4 (CI 9.2–9.6)

Lastly, the proportion of deaths in other communal establishments experienced steady increases across all groups. Trend for time analyses identified that these increases were significant for those from the UK (average annual change in percentage point 0.65 per year, 95% CI 0.45–0.85, p<0.001) and from Ireland (average annual change in percentage point 0.59, p = 0.003, 95% CI 0.27–0.91, p = 0.003).

**Figure 3:**
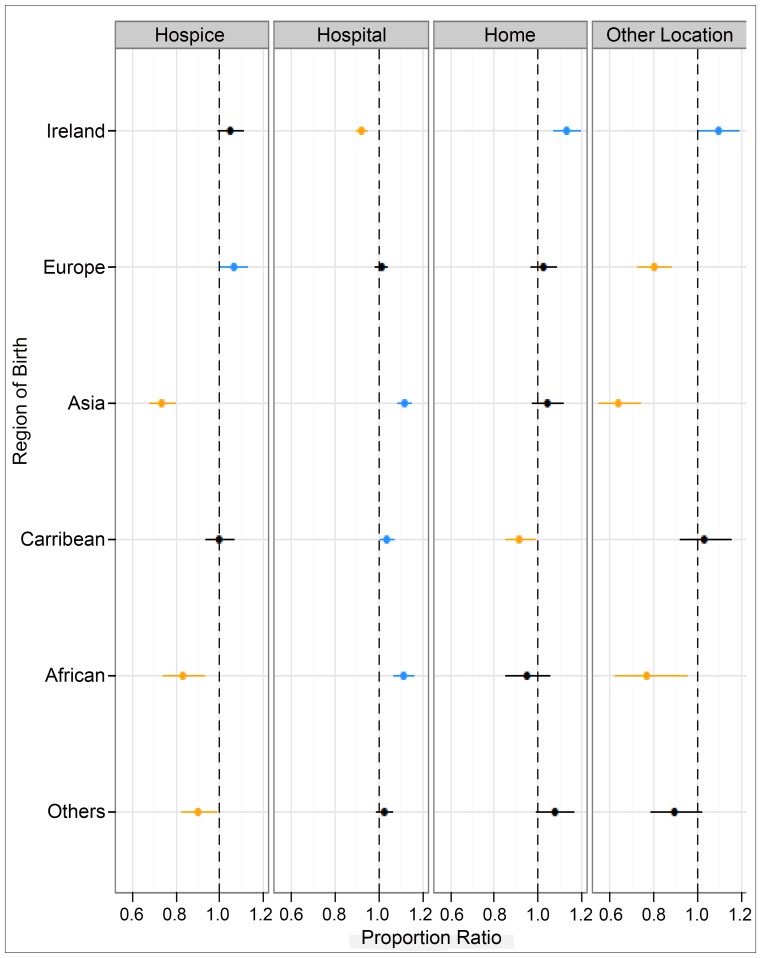
Adjusted proportional ratios (PR) for (i) deaths in hospice, (ii) deaths in hospital settings, (iii) death at home and, (iv) deaths in other communal establishments versus deaths in all other settings.

Poisson regression modelling provided a better or equal overall model performance than alternative models. Following adjustment for sex, age, marital status, cancer cause and deprivation, table 3 and figure 3 demonstrate that deaths in hospital settings were less likely among those born in Ireland (PR 0.92 [0.90–0.95]), but more likely for those born in Asia, including China (PR 1.12 [1.08–1.15]) and Africa (PR 1.11 [1.07–1.16]). Compared to all other settings, deaths in hospice settings were significantly less likely among those born in Asia, including China (PR 0.73 [0.68–0.80]), Africa (PR 0.83 [0.74–0.93]) and ‘other’ locations (PR 0.90 [0.82–0.98]). Deaths in decedents' own homes were significantly less likely among those born in the Caribbean (PR 0.91 [0.85–0.98]) and more likely among those born in Ireland (PR 1.13 [1.07–1.19]), compared to all other locations. Finally, deaths in other communal establishments including nursing and residential care homes were more likely for those born in Ireland (PR 1.01 [1.01–1.19]) and less likely for those born in Europe (PR 0.80 [0.73–0.88]), Asia, including those from China (PR 0.64 [0.55–0.74]) and Africa (PR 0.77 [0.62–0.95]).

**Table 3:** Adjusted proportional regression (* PR) ratios for (a) deaths in hospice (b) deaths at hospital (c) deaths in own residence, and (d) deaths in communal establishment versus all other locations for the period 2001–2006: Adjusted for sex, age, year, marital status, IMD quintile, and all cancer causes.

		PR *	(unadjusted 95% CI)	PR *	(adjusted 95% CI)
**Hospice v/s all other locations**	UK	1.00	(1.00–1.00)	1.00	(1.00–1.00)
	Ireland	1.15	(1.07–1.23)	1.05	(0.99–1.11)
	Europe	1.08	(1.00–1.16)	1.06	(1.00–1.13)
	Asia	0.73	(0.66–0.81)	0.73	(0.68–0.80)
	Caribbean	1.12	(1.04–1.22)	1.00	(0.93–1.06)
	African	0.91	(0.79–1.04)	0.83	(0.74–0.93)
	Others	0.93	(0.84–1.04)	0.90	(0.82–0.98)
**Hospital v/s all other locations**	UK	1.00	(1.00–1.00)	1.00	(1.00–1.00)
	Ireland	0.93	(0.90–0.96)	0.92	(0.90–0.95)
	Europe	1.01	(0.99–1.04)	1.01	(0.99–1.04)
	Asia	1.13	(1.10–1.16)	1.12	(1.08–1.15)
	Caribbean	1.06	(1.03–1.09)	1.03	(1.01–1.07)
	African	1.12	(1.07–1.17)	1.11	(1.07–1.16)
	Others	1.03	(0.99–1.07)	1.02	(0.99–1.06)
**Own Residence v/s all other locations**	UK	1.00	(1.00–1.00)	1.00	(1.00–1.00)
	Ireland	1.13	(1.07–1.19)	1.13	(1.07–1.19)
	Europe	1.04	(0.98–1.10)	1.02	(0.96–1.09)
	Asia	1.13	(1.06–1.21)	1.04	(0.98–1.11)
	Caribbean	0.90	(0.84–0.96)	0.91	(0.85–0.98)
	African	1.03	(0.93–1.14)	0.95	(0.85–1.05)
	Others	1.09	(1.00–1.18)	1.08	(0.99–1.16)
**Other Communal v/s all other locations**	UK	1.00	(1.00–1.00)	1.00	(1.00–1.00)
	Ireland	0.91	(0.84–0.99)	1.10	(1.01–1.19)
	Europe	0.77	(0.70–0.85)	0.80	(0.73–0.88)
	Asia	0.47	(0.40–0.54)	0.64	(0.55–0.74)
	Caribbean	0.73	(0.66–0.82)	1.03	(0.92–1.15)
	African	0.50	(0.41–0.63)	0.77	(0.62–0.95)
	Others	0.80	(0.70–0.92)	0.90	(0.79–1.02)

## Discussion

Policy and law across Europe, and much of the world, requires the health-care needs of different ethnic groups to be fairly met [30]. Moreover, it is a basic human right [31]. The propelling motive is the quest for equity and equality in health status and health outcomes. This includes end of life care. We made use of death registration data to examine the relationship between decedent's country of origin and location of their death. We identify that whilst there was a decline in the number of hospital deaths and a corresponding increase in the number of home deaths across the study period for all those aged ≥65 years, the gaps according to country of birth have not narrowed over time. Secondly, when compared to all other settings, deaths in a hospice were far less common for those born outside of the UK and deaths in a hospital setting were more common.

### Differences that make a difference

Our analysis identified that, during the study period, those dying from cancer that were born in Asia including China, Africa, and ‘other’ geographical locations, were significantly less likely than their UK born equivalents to die in a hospice. Moreover, those born in the Caribbean were significantly less likely to die at home than those born in the UK or Ireland. Hospital deaths were more likely among those born in Asia including China and Africa, compared to those from other regions in the world. It was also identified that, dying in other communal establishments, principally residential care and nursing homes were more likely among those born in Ireland than other groups. None of the findings were altered by adjustment for other important factors, including deprivation and cancer cause. To date, most studies exploring ethnicity as an explanatory factor in place of care or death have been carried out in the USA and have identified that White Caucasian individuals were more likely than other ethnic groups to access hospice services [32] [33] and less likely to die in hospital [34]. However, elsewhere, significant disparities between ethnic groups have not been evident [35].

Only one other UK study of cancer registration data has examined the relationship between place of death and ethnicity, this study was limited by the quality of the data in which ethnicity remained unknown for a third of registrations in the database [28]. The authors identified that among 68,804 cases, deaths in a hospice were less likely for those who were born in Asia (Pakistan or Bangladesh); this supports the findings from our study. Coupland and Madden et al. (2011) found home deaths were less likely amongst some BAME groups including Black Africans, Caribbeans and Chinese. This is in contrast to our analysis, which found that only those born in the Caribbean were less likely to die at home than other groups. This finding also differs markedly from a USA-based study in which black individuals were identified as more likely to die at home [36]. Our analysis identified that those who were born in Africa, the Caribbean or Asia were all more likely to die in a hospital, than those born in the UK or Europe. This finding is in contrast to a study in the USA where Black Americans were observed as dying at home, in similar proportions to their white peers [37] and further disparities are evident with another USA-based study where African American individuals were more likely to die in a hospital than White individuals [38].

### Preferences for place of care and place of death

Enabling people to make choices and achieve their preferences for end of life care is a core value enshrined in the UK's NHS *End of Life Care Strategy* (2008) and in the USA hospice benefit programme [38] [39] [40]. These both emphasisethe importance of ensuring terminally ill adults are able to choose where they die. Studies on end of life preferences in the UK, Europe and the rest of the world have found that most people prefer to die at home [14], [41]. Research among BAME groups living in the UK on preferred place of care and death, however, is still sparse and has been parochial in nature and qualitative, therefore remaining difficult to generalise to other settings and populations [42]. We found that home deaths in London across all groups aged ≥65 years increased, although some groups, for example those born in the Caribbean were less likely to die at home than in other locations. No study, other than this one, has comprehensively explored ethnicity (or a proxy for ethnicity) as an explanatory factor in place of death, on this scale, using ONS death registry data.

### Opportunities for health gain during life and at death

In the UK, health care provision, including specialist palliative care provided within the independent sector, is free-at-the-point of delivery. However, access to and availability of specialist palliative care services has repeatedly been shown to be variable and inequitable throughout the country [43]; dying in a hospice remains the preserve of certain groups over others [44]. As palliative care services in the UK reach a higher level of maturity, there has been increasing attention on the part of policy makers to ensure equality of access. Efforts to better improve health services for socially excluded groups [45] and care at the end-of-life in particular, are now more common; *Help the Hospices*' *Widening Access Project (WAP)* [46], and London-specific project *Social Action for Health* (SAfH) [47] are recent initiatives aiming to specifically improve up-take of palliative care services, although they remain unevaluated. Importantly, our data cannot demonstrate to what extent decedents made use of specialist palliative care services, although we can make some reasonable assumptions about the care received by those who died in a hospice. Furthermore, we are not able to determine the preferred place of care during the final phase of illness. Nevertheless, our findings contribute to a growing body of evidence that suggests a significant number of people, including older people [48] and materially deprived [11], may miss out on important aspects of palliative and end-of-life care including the option to die in a hospice or at home [10]. These findings are even more significant when contrasted against evidence that the use of primary care services by patients from BAME and lower socio-economic groups is either equivalent, or higher, than their white British, or less materially deprived counterparts, even after adjustment for crude measures of need [49]. Possible reasons for disparities in service use include (i) lack of awareness and knowledge of palliative care and related services [11]; (ii) referral patterns to specialist palliative care [50]; (iii) lack of understanding amongst professionals about exactly which patients to refer and when [10]; (iv) gate-keeping by services [51]; (v) complex linguistic and communication barriers [50]; (vi) preferences including for more aggressive or curative care at the end-of-life, or a cultural mistrust of end-of-life care [52]; and (vii) strong religious and familial support systems [53].

Although there is a general lack of data about people from BAME communities at the end of life, what is available demonstrates that for some groups the experience of disadvantage during their lives [54], is also evident in death. Minority ethnic communities may also experience disproportionate levels of material deprivation and other forms of social disadvantage. Additionally, people from minority ethnic communities may also experience overt and inadvertent racial discrimination at an individual and institutional level [55].

### Study strengths and weaknesses

Death registration has a long history of being used as a health indicator and monitoring tool for public health policy. A major strength of our study is that of completeness: death certificates allow the description of patterns within a whole population, rather than just for a sample [23] [56]. To our knowledge, this is the first attempt to test the relationship between country of birth and place of death using the death registration database. Given the exploratory nature of the investigation, we did not undertake multiple testing adjustment, but applied a more liberal approach to report our findings, to curtail the likelihood of Type II error [57].

There are, however, a number of weaknesses to this study that affect the inferences that can be made from the findings we present. Firstly, our reliance on country of birth as a proxy for ethnicity is open to criticism [58]. The decision to use a proxy indicator for ethnicity [4] [6] [24] [25], as has been done elsewhere in end-of-life care research [26] [59], was driven by the current omission of ethnicity within the ONS death registry dataset. Another approach to further comprehendhealth variations across different ethnic groups would be to use hospital episodes statistics (HES). However, whilst the completeness of HES has improved in recent years [60], it still does not fully represent all patient episodes [61]; furthermore, the Thames Cancer Register has been used to assess the completeness of HES ethnicity data and identified that between one fifth (22.7%) [62] to one third (32.2%) [28] of all patients in the dataset, had no recorded ethnicity. Computerised probability matching techniques can link hospital discharge and mortality to census records, which may promise potential solutions to this problem [63]. In the meantime, poor quality or absence of recording of ethnicity data leads to ‘social invisibility’ of paradoxically growing populations [25]. Efforts to collect ethnicity data should therefore be encouraged across all healthcare settings. Moreover, future research should explore where the difficulties collecting ethnicity information lie, whether with patients, healthcare professionals or the recording procedure, and how such problems can be overcome [62].

Secondly, whilst we have conducted analysis on a complete data set for all cancer deaths across London over a nine year period, the number of people in the dataset who were born in some countries was still very small. As a consequence, some countries had to be grouped together. For example, those born in China were amalgamated into the larger Asian population in order to be included in the proportional regression analyses. These artificial groupings conflate a wide range of ethnic identities including a variety of beliefs, identities, cultures, notions of social support, history and religions [2].

Thirdly, a comparison of USA studies with the UK presents challenges due to important differences in the organisation of healthcare systems, palliative care provisions and in the actual classification of different ethnic groups [2]. For example, many of the studies in the USA considered the general use of hospice services, which can include both domiciliary and inpatient care. In the UK, however, deaths within a hospice refer only to deaths within that setting. In the UK, it is therefore not possible to identify decedents who died at home who were also in receipt of, hospice or community specialist palliative care.

Finally, as a result of basing our study on routine statistics, we are unable to explore other relationships that may also govern our findings. These would include data on the availability of services and the social context of decedents' lives; a number of complex inter-related factors including size and level of social support, marital status, religious beliefs beyond ethnicity and country of birth- these have all been identified as influencing end of life-related decisions [53] including preferred for place of care and death [64]. To examine these complex factors would require a study of a different kind.

## Conclusions

This is the first population-based UK study using death registration data to examine variations in place of death from cancer across different ethnic groups. We found that location of death varies according to decedents' country of birth. People who were born in Asia and Africa were significantly less likely than those from the UK or Ireland to die in a hospice. People born in the Caribbean were significantly less likely to die at home than those born in the UK. Decedents born in Asia, Africa and the Caribbean were all significantly more likely than those from UK to die in a hospital. We do not know to what extent these variations are a result of differing preferences for place of death, which may be influenced by culture. Nor can we accurately identify which factors related to diagnosis, personal, environmental or service-related circumstances may prevent certain groups from accessing specialist palliative care. More detailed prospective studies are urgently required to understand this. Such knowledge will help in the development of co-ordinated strategies that aim to narrow the gaps between patient and family-centred preferences for, and actual, location of death.
