# Correction: Does Ethnicity Affect Where People with Cancer Die? A Population-Based 10 Year Study

**DOI:** 10.1371/journal.pone.0106019

**Published:** 2014-08-13

**Authors:** The PLOS ONE Staff

The third author's name is incorrectly missing her middle initial. The correct name is: Joanna M Davies. The correct citation is: Koffman J, Ho YK, Davies JM, Gao W, Higginson IJ (2014) Does Ethnicity Affect Where People with Cancer Die? A Population-Based 10 Year Study. PLoS ONE 9(4): e95052. doi:10.1371/journal.pone.0095052
